# AIPpred: Sequence-Based Prediction of Anti-inflammatory Peptides Using Random Forest

**DOI:** 10.3389/fphar.2018.00276

**Published:** 2018-03-27

**Authors:** Balachandran Manavalan, Tae H. Shin, Myeong O. Kim, Gwang Lee

**Affiliations:** ^1^Department of Physiology, Ajou University School of Medicine, Suwon, South Korea; ^2^Institute of Molecular Science and Technology, Ajou University, Suwon, South Korea; ^3^Division of Life Science and Applied Life Science (BK21 Plus), College of Natural Sciences, Gyeongsang National University, Jinju, South Korea

**Keywords:** AIPpred, anti-inflammatory peptides, random forest, hybrid features, parameter optimization

## Abstract

The use of therapeutic peptides in various inflammatory diseases and autoimmune disorders has received considerable attention; however, the identification of anti-inflammatory peptides (AIPs) through wet-lab experimentation is expensive and often time consuming. Therefore, the development of novel computational methods is needed to identify potential AIP candidates prior to *in vitro* experimentation. In this study, we proposed a random forest (RF)-based method for predicting AIPs, called AIPpred (AIP predictor in primary amino acid sequences), which was trained with 354 optimal features. First, we systematically studied the contribution of individual composition [amino acid-, dipeptide composition (DPC), amino acid index, chain-transition-distribution, and physicochemical properties] in AIP prediction. Since the performance of the DPC-based model is significantly better than that of other composition-based models, we applied a feature selection protocol on this model and identified the optimal features. AIPpred achieved an area under the curve (AUC) value of 0.801 in a 5-fold cross-validation test, which was ∼2% higher than that of the control RF predictor trained with all DPC composition features, indicating the efficiency of the feature selection protocol. Furthermore, we evaluated the performance of AIPpred on an independent dataset, with results showing that our method outperformed an existing method, as well as 3 different machine learning methods developed in this study, with an AUC value of 0.814. These results indicated that AIPpred will be a useful tool for predicting AIPs and might efficiently assist the development of AIP therapeutics and biomedical research. AIPpred is freely accessible at www.thegleelab.org/AIPpred.

## Introduction

Inflammatory responses are tightly controlled under normal conditions and are essential for the initiation of protective immunity (Medzhitov, 2008; Basith et al., 2011b, 2012). When these responses occur in the absence of infection or persist after their routine function, these processes become pathological, resulting in chronic inflammation and autoimmune disorders, including neurodegenerative disease, rheumatoid arthritis, asthma, psoriasis, diabetes, and multiple sclerosis (Asadullah et al., 2002; Balague et al., 2009; Murdoch and Lloyd, 2010; Steinman et al., 2012; Patterson et al., 2014). The current therapy for inflammatory and autoimmune disorders involves the use of non-specific anti-inflammatory drugs and other immunosuppressants, which are often associated with potential side effects, such as ineffectiveness against inflammatory disorders and induction of a higher risk of infectious diseases (Tabas and Glass, 2013).

Because peptide-based therapy has several advantages over small molecules owing to their high specificity and minimal toxicity under normal conditions, anti-inflammatory peptides (AIPs) act as potent therapeutic agents for inflammatory and autoimmune disorders (de la Fuente-Nunez et al., 2017; Wu et al., 2017). For example, chronic nasal administration of human amyloid-β peptide (40 amino acid residues) in an Alzheimer’s disease mouse model resulted in reduced deposition of amyloid-β, which is a pathological marker of Alzheimer’s disease, microgliosis, astrocytosis, and neuritic dystrophy in the brain (Weiner et al., 2000). Vasoactive intestinal peptide reduces inflammation in rheumatoid arthritis by altering the immune response to reduce cytokine production in CD4^+^ T cells (Delgado et al., 2001). RDP58, a synthetic decapeptide, effectively inhibits the production of inflammatory cytokines, such as tumor necrosis factor-α, interferon (IFN)-γ, IL-2, and IL-12, as well as the infiltration of inflammatory cells associated with urothelial inflammatory response in an *in vivo* model of lipopolysaccharide-induced cystitis (Boismenu et al., 2002; Gonzalez et al., 2005). Furthermore, AIPs act as potent candidates for cancer prevention and therapy because inflammation is closely linked to cancer (Rayburn et al., 2009).

Although AIPs specifically bind to the receptor and activate signaling cascades in cells, experimental identification and development of novel AIPs represent extremely expensive and often time-consuming processes. Therefore, the development of sequence-based computational methods is necessary to allow the rapid identification of potential AIP candidates prior to their synthesis. It should be noted that the prediction methods prior to synthesis would help a number of previous design studies (Geetha et al., 2005; Grieco et al., 2005; Park et al., 2009). To this end, Gupta et al. (2017b) developed a support vector machine (SVM)-based method to predict AIPs using trinucleotide composition and motif features. This represents the first and only method available for AIP prediction, and although this method has stimulated further development in this area, additional work is needed for the following reasons: (*i*) with the steadily increasing number of anti-inflammatory epitopes or peptides in the Immune Epitope Database (IEDB), it is necessary to develop more accurate prediction methods with a larger benchmark dataset. (*ii*) The feature space used by the existing method is incomplete; hence, additional potent features are needed to be characterized. Owing to these deficiencies, other methods are necessitated to accurately predict AIPs by taking advantage of machine learning (ML) algorithms and informative feature extraction based on high-quality benchmarking datasets.

In this study, we developed a random forest (RF)-based method to predict AIPs, called AIPpred (AIP predictor from primary amino acid sequences), in which optimal features were selected using a feature selection protocol, which has been implemented in addressing various biological problems (Manavalan and Lee, 2017; Manavalan et al., 2017b, 2018). First, we studied the contribution of individual composition [amino acid composition (AAC), amino acid index (AAI), dipeptide composition (DPC), chain-transition-composition (CTD), and physicochemical properties (PCP)] in AIP prediction. Since the DPC-based model significantly outperformed other composition-based models, we applied a feature selection protocol on DPC and identified the optimal features. In addition to AIPpred, we also developed SVM, extremely randomized tree (ERT), and *k*-nearest neighbors (*k*-NN)-based methods. It is to be noted that, when objectively evaluated using an independent dataset, AIPpred displayed superior performance compared to the currently available method AntiInflam and 3 other ML methods (ERT, SVM, and *k*-NN) developed in this study.

## Materials and Methods

For the development of our method, we followed the 5 guidelines (Chou, 2011) mentioned in a series of recent publications (Chen W. et al., 2016; Chen et al., 2017; Feng et al., 2017; Liu et al., 2017) on new peptide-prediction methods that could be easily accessed by both experimentalists and theoretical scientists: (i) construct a valid benchmarking dataset to train and test the prediction model; (ii) formulate the biological-sequence samples with an effective mathematical expression truly reflecting their intrinsic correlation with the target to be predicted; (iii) introduce or develop a powerful algorithm (or engine) to operate the prediction; (iv) properly perform cross-validation tests to objectively evaluate the anticipated accuracy of the predictor; and (v) establish a user-friendly web server for the predictor that is accessible to the public. Below, we describe in detail the application of each of these steps.

### Dataset Construction

To build a classification model, a well curated dataset is required. Hence, we extracted experimentally validated positive and negative linear peptides or epitopes from the IEDB (Zhang et al., 2008; Fleri et al., 2017). A peptide induced any one of the anti-inflammatory cytokines [IL-10, IL-4, IL-13, IL-22, TGFβ, and IFN-α/β] in T-cell assays of human and mouse (Marie et al., 1996), was considered positive. Similarly, linear peptides testing negative for anti-inflammatory cytokines were considered negative. To generate a non-redundant (nr) dataset, we eliminated redundant peptides using CD-HIT (Huang et al., 2010) by applying a sequence identity threshold of 0.8, indicating that sequence identity between any two sequences greater than 80% is discarded. Using a more stringent criterion, such as 30 or 40%, as imposed in (Gupta et al., 2013; Ding et al., 2014; Chen X-X. et al., 2016), could improve the credible reliable of the model. However, in this study we do not use such a stringent criterion, because the currently available data does not allow it. Otherwise, the number of samples for some subsets would be insufficient for statistical significance.

Finally, we obtained an nr dataset of 1678 AIPs and 2,516 non-AIPs, whose size is ∼2-fold bigger than the dataset used in the previous method (i.e., AntiInflam) (Gupta et al., 2017b). From this nr dataset, 80% of the data was randomly selected as the benchmarking dataset (i.e., 1258 AIPs and 1,887 non-AIPs) to develop a prediction model, whereas the remaining 20% was considered the independent dataset (i.e., 420 AIPs and 629 non-AIPs).

### Feature Extraction

We formulated the AIP-prediction task as a binary classification problem (AIP or non-AIP) and solved it using RF, SVM, *k*-NN, and ERT algorithms. An important aspect of this process involves the extraction of a set of relevant features. Therefore, we used AAC, AAI, DPC, PCP, and CTD, whose definitions are briefly discussed in the following subsections.

#### Amino Acid Composition

AAC is defined as the fraction of each amino acid in the given peptide sequence, and it was calculated using the following equation (1).

(1) $$\mathrm{AAC}(i)=\frac{Frequency of amino acid(i)}{Length of the peptide}$$

where *i* can be any one of the 20 natural amino acids. AAC has a fixed length of 20 features.

#### Amino Acid Index

The AAIndex database contains amino acid indices of various physicochemical and biochemical properties (Kawashima et al., 2008). Saha et al. (2012) classified these amino acid indices into eight clusters, and the central indices of each cluster were named as high-quality amino acid indices: BLAM930101, BIOV880101, MAXF760101, TSAJ990101, NAKH920108, CEDJ970104, LIFS790101, and MIYS990104. We averaged eight high-quality amino acid indices (i.e., a 20-dimensional vector) as an input feature.

#### CTD

The CTD feature was introduced by Dubchak et al. (1995) for predicting protein-folding classes. Thereafter, it was successfully applied in various sequence-based classification algorithms (Cai et al., 2003; Magnan et al., 2009; Wang et al., 2016; Hasan et al., 2017). CTD represents the distribution of amino acid patterns along the primary sequence, based on their physicochemical or structural properties. There are seven physiochemical properties, including hydrophobicity, polarizability, normalized van der Waals volume, secondary structure, polarity, charge and solvent accessibility.

All amino acids are divided into three groups: polar, neutral and hydrophobic. C consists of three percentage composition values for a given peptide: polar, neutral and hydrophobic. T consists of the percentage frequency of a polar followed by a neutral residue or of a neutral by a polar residue. It may also consist of a polar, followed by a hydrophobic residue or a hydrophobic followed by a polar residue. It may also consist of a neutral, followed by a hydrophobic or a hydrophobic, followed by a neutral residue. D consists of five values for each of the three groups. It measures the chain length, within which the first, 25, 50, 75, and 100 % of the amino acids of a specific property are located. There are three descriptors and 3(C) + 3(T) + 5 × 3(D) = 21 descriptor values for a single amino acid attribute. Consequently, seven different amino acid attributes produce a total of 7 × 21 = 147 features.

#### Dipeptide Composition

DPC is defined as the total number of dipeptides normalized against 400 possible dipeptides in the given peptide sequence and was calculated using the following equation (2):

(2) $$\text{DPC}(i)\text{=}\frac{\text{Total number of dipeptides}(i)}{\text{Total number of all possible dipeptides′}}$$

where *i* can be any one of the 400 possible dipeptides. DPC has a fixed length of 400 features.

#### Physicochemical Properties

Frequencies of the following features are directly computed from the sequence consisting of: (1) hydrophobic (i.e., F, I, W, L, V, M, Y, C, A); (2) hydrophilic (i.e., R, K, N, D, E, P); (3) neutral (i.e., T, H, G, S, Q); (4) positively charged (i.e., K, H, R); (5) negative-charged (i.e., D, E); (6) turn-forming residues fraction (i.e., (N + G + P + S)/n, where *n* = sequence length); (7) absolute charge per residue (i.e., 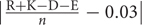); (8) molecular weight and (9) aliphatic index (i.e., (A+2.9V+3.9I+3.9L)/n).

### Machine Learning Methods

In general, the major advantage of the ML method is that it can identify the hidden relationship between the input features and the objective values in a complex dataset, which will be helpful for accurate prediction (Cao et al., 2014, 2016a,b, 2017; Manavalan et al., 2014, 2017a, 2010b; Cao and Cheng, 2016; Manavalan and Lee, 2017). In this study, we used 4 different ML methods (ERT, RF, *k*-NN and SVM) to develop their prediction models using benchmarking datasets. The description of these methods is provided as follows.

#### Random Forest

Breiman (2001) proposed RF as an ensemble technique to perform predictions using 100s or 1000s of independent decision trees. RF is one of the most popular ML methods and is used as a computational approach to numerous biological problems. Detailed descriptions of the RF algorithm have been provided in earlier studies (Lee et al., 2013, 2015; Manavalan et al., 2014). In the RF algorithm, the number of trees (*ntree*), variables randomly chosen at each node split (*mtry*), and the minimum number of samples required to split an internal node (*nsplit*) are the 3 most influential parameters that require optimization. We optimized these parameters using a grid search within the following ranges: *ntree* from 50 to 1,000, with a step size of 20; *mtry* from 1 to 7, with a step size of 1; and *nsplit* from 2 to 10, with a step size of 1.

#### Extremely Randomized Tree

Geurts et al. (2006) proposed ERT as an ensemble technique utilizing hundreds of independent decision trees to perform classification. Although the ERT algorithm is similar to that of RF, the major differences are that ERT uses the entire training sample instead of a bootstrap sample (RF) to construct a tree, and the ERT splitting criterion is random, whereas RF uses information gain measured by the Gini impurity. Furthermore, the parameter-optimization procedure is the same as that used for the RF method.

#### Support Vector Machine and *k*-Nearest Neighbors

Descriptions of SVM and *k*-NN along with their optimization procedures have been provided in earlier studies (Manavalan et al., 2015, 2017a, 2010b; Manavalan and Lee, 2017). We followed the same procedures in this study.

### Evaluation Metrics

To compare the prediction methods, we used the following five metrics: sensitivity, specificity, accuracy, Mathews’ correlation coefficient (MCC), and the area under receiver operating characteristics (ROC). All these metrics are commonly used in the literature to measure the quality of binary classification (Porto et al., 2017b).

(3) $$\{\begin{array}{c} Sensitivity=\frac{\mathrm{TP}}{\mathrm{PS}},\\ \mathrm{Specificity}=\frac{\mathrm{TN}}{\mathrm{NS}},\\ \mathrm{Accuracy}=\frac{TP+TN}{PS+NS},\\ MCC=\frac{1-(\frac{\mathrm{FN}}{\mathrm{PS}}+\frac{\mathrm{FP}}{\mathrm{NS}})}{\sqrt{\left(1+\frac{\mathrm{FP}-\mathrm{FN}}{\mathrm{PS}})(1+\frac{\mathrm{FN}-\mathrm{FP}}{\mathrm{NS}}\right)}}, \end{array}$$

where TP, FN, TN, and FP respectively represent the number of true positive, false negative, true negative and false positive. PS and NS respectively represent the total number of sequences in the positive set (AIPs) and negative set (non-AIPs).

AUC is the area under the ROC curve, representing the relationship between TP rate and FP rate of the model. The AUC is an indicator of the performance quality of the binary classifier.

### Development of a Prediction Server

We developed an online prediction server using hypertext mark-up language and JavaScript, with a Python script executed in the background upon submission of peptide sequences in the FASTA format. Users can submit single or multiple sequences containing only standard amino acid residues in FASTA format. The AIPpred web server outputs the predicted class along with probability values for the given peptide sequence.

## Results

### Compositional and Positional Information Analysis

**FIGURE 1:**
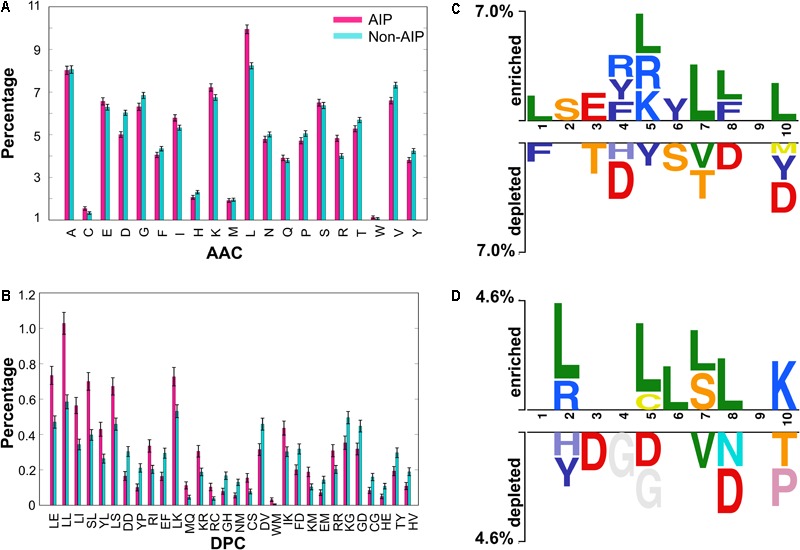
Compositional and positional preference analysis. **(A)** and **(B)** respectively represent the amino acid and dipeptide preferences between AIPs and non-AIPs. **(B)** Significant differences in top-30 dipeptides are shown. **(C,D)** Represent positional conservation of ten residues at N- and C-terminal between AIPs and non-AIPs, respectively, generated using two sample logos.

We performed compositional analysis using the combined dataset (i.e., benchmarking and independent). AAC analysis revealed that average composition of certain residues, including Arg, Leu and Lys, were dominant in AIPs. However, Gly, Asp, Val, Tyr and Pro were dominant in non-AIPs (Welch’s *t*-test; *P* ≤ 0.05) (**Figure 1A**). Furthermore, DPC analysis revealed that 19% of dipeptides differed significantly between AIPs and non-AIPs (Welch’s *t*-test; *P* ≤ 0.05). Of these, the top-10 most abundant dipeptides in AIPs and non-AIPs were LL, SL, LE, LI, LS, LK, YL, IK, RI and KR, and DV, KG, DD, EF, GD, FD, YP, TY, GH and HV, respectively (**Figure 1B**). These results suggest that the most abundant dipeptides in AIPs consist primarily of pairs of aliphatic-aliphatic, positively charged-positively charged or -aliphatic, and hydroxyl group-aliphatic or -aromatic amino acids. However, the most abundant dipeptides in the non-AIPs were negatively charged-negatively charged or -aliphatic, and positively charged-negatively charged amino acids. Overall, significant differences observed in compositional analysis could be incorporated into ML algorithms to improve prediction performances. Hence, we considered them as input features.

To understand the positional information of each residue, a sequence logo of the first ten residues from the N- and the C-terminal of AIPs and non-AIPs were generated using two sample logos. To test their statistical significance, the height of the peptide logos were scaled (*t*-test by P < 0.05). At the N-terminal, we found that, compared to other amino acids, R, at positions 4 and 5; L, at positions 1, 5, 7, 8, and 10; F, at positions 4 and 8; and Y, at positions 4 and 6 were significantly overrepresented. Alternatively, negatively charged residue D, at positions 4, 8, and 10; and S/T, at positions 3, 6, and 7 were significantly underrepresented (**Figure 1C**). No significant amino acids were found at enriched position 9 or the depleted positions 2 and 9. C-terminal R/K, at positions 2 and 10; and L, at positions 2, 5, 6, 7, and 8 were significantly overrepresented. Alternatively, negatively charged residues D, at positions 3, 5, and 8 and G, at positions 4 and 5 were significantly underrepresented (**Figure 1D**). No significant amino acids were found at enriched position 1, 3, 4, and 9 or the depleted positions 1, 6, and 9. These results suggest that comparatively residues, L and R/K, are preferred in AIPs. This is consistent with the AAC analysis observation. Furthermore, positional preference analysis will be helpful for experimenters who design *de novo* AIPs and substitute amino acids at particular positions to make the peptides more effective.

### The Overall Framework of the AIPpred Approach

**FIGURE 2:**
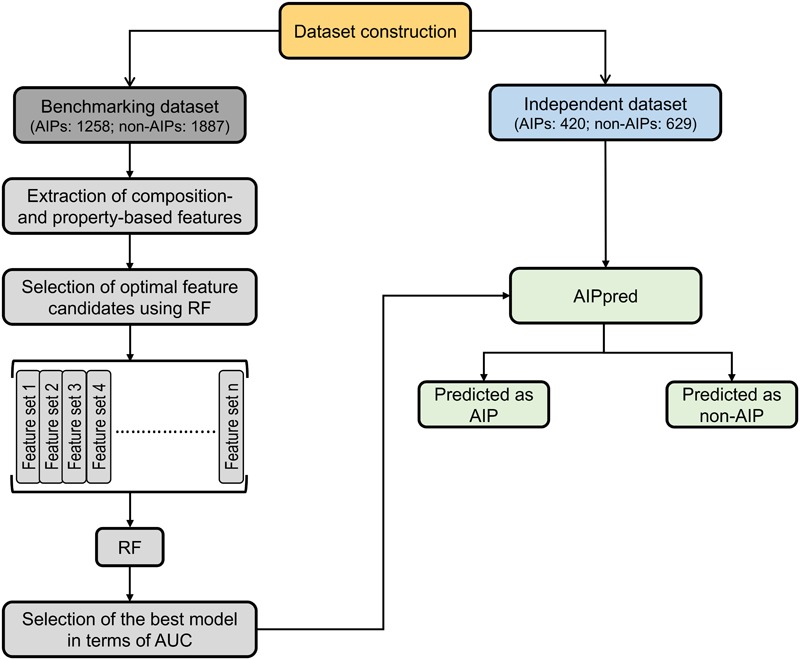
Overall framework of the proposed predictor. AIPpred development involved the following steps: (1) dataset curation, (2) feature extraction, (3) generation of different feature sets and development of prediction models using RF algorithm, and (4) model selection.

The overall framework of AIPpred is shown in **Figure 2**. It consists of the following 4 stages: (1) construction of a nr benchmarking dataset of 3,145 peptides (1,258 AIPs and 1,887 non-AIPs) and an independent dataset of 1,049 peptides (420 AIPs and 629 AIPs); (2) extraction of various features from peptide sequences, including AAC, AAI, CTD, DPC, and PCP; (3) systematic evaluation of individual composition and generation of 35 different feature sets based on the feature importance scores (FISs) computed using the RF algorithm. These different feature sets were inputted to RF, and their respective prediction models were built; and (4) selection of the best model.

### Performances of RF Models Based on Individual Composition

**FIGURE 3:**
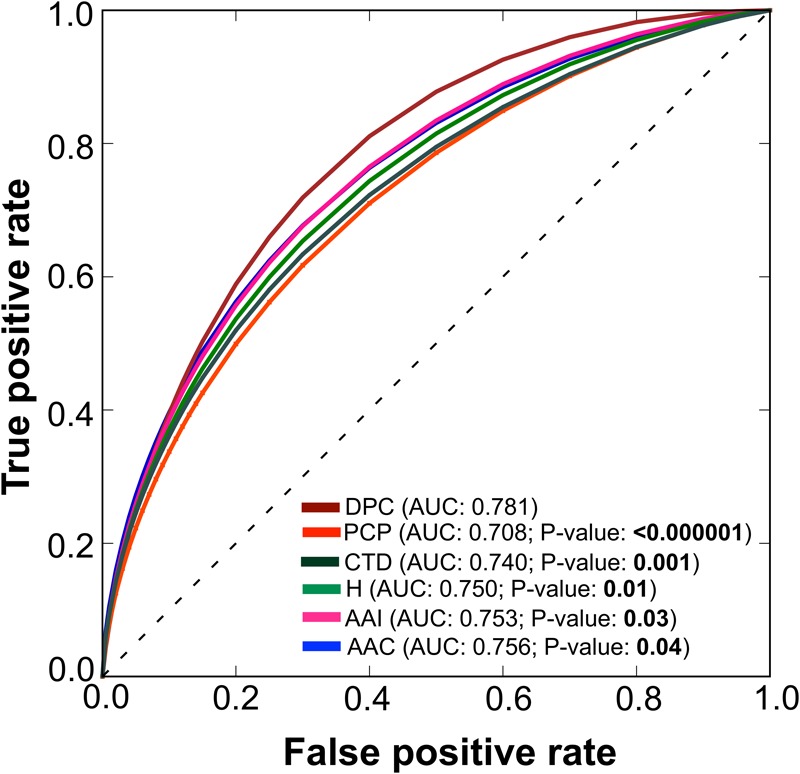
A graphical illustration to show the performance of various composition-based RF models in terms of ROC curves obtained from the 5-fold cross-validation. A pairwise comparison of AUC between DPC and the other composition-based model was computed using a two-tailed *t*-test (Hanley and McNeil, 1982). A *P* ≤ 0.05 indicates a statistically meaningful difference between DPC and the selected model (shown in bold).

To test the effectiveness of individual composition in AIP prediction, we inputted each composition separately to RF and developed their corresponding prediction models, as well as a model based on hybrid features (linear combination of individual composition). The performance of these models is shown in **Figure 3**. At a *P*-value threshold of 0.05, the DPC-based model significantly outperformed 4 other individual (PCP, AAI, CTD, and AAC) composition-based models and hybrid (H) models. Hence, we considered only the DPC-based model for further analysis. In the DPC-based model, all possible dipeptides are not equally important for the trained model performance. The inclusion of irrelevant dipeptides during training might reduce model performance. Therefore, a feature selection paradigm is essential to remove irrelevant dipeptides and consequently improve the prediction performance.

### Feature Selection Protocol

**FIGURE 4:**
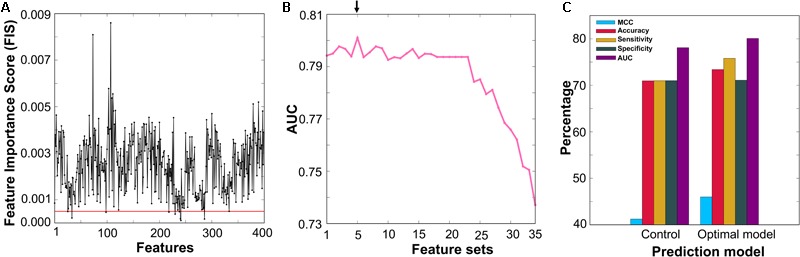
**(A)** The *x*- and *y*-axes represent each feature and its feature importance score (FIS), respectively. We applied a FIS cut-off of ≥0.0005 and selected 391 features (above the red line) as optimal feature candidates. **(B)** RF-based model performance in terms of AUC with respect to the different feature set. The final selected optimal model is shown with an arrow. **(C)** A comparison between the optimal model and control.

The feature selection protocol employed in this study is the same as the one used in recent studies (Manavalan et al., 2017b; Manavalan and Lee, 2017). First, we applied the RF algorithm and estimated the FISs of 400 dipeptides in distinguishing AIPs and non-AIPs. In short, all features were inputted to the RF, and 5-fold cross-validation was carried out using the benchmarking dataset. For each round of cross-validation, we built 10,000 trees, and the number of variables at each node was chosen randomly from 1 to 50. The average FISs from all the trees are shown in **Figure 4A** and Supplementary Table S1, where ∼36% of the features (FIS ≥ 0.003) contributed significantly to AIP prediction. Second, we excluded 9 features that have a low FIS (less than 0.0005) and generated 35 different feature sets based on FIS cut-off (0.0005 ≤ FIS ≤ 0.0039, with a step size of 0.0001) with the remaining 391 features. In general, the optimal feature set lies in between a large number of features that contain considerable irrelevant information and a small number of only important features (describing a part of AIP properties). The 35 different feature sets generated have a feature size ranging from 49 to 391. Basically, we eliminated less important features in a step-wise manner. Finally, we inputted each set into the RF algorithm and optimized ML parameters (*mtry, ntree*, and *nsplit*) by 5-fold cross-validation on the benchmarking dataset. To check the robustness of the model performance, we carried out 5-fold cross-validation 10 times by randomly portioning the benchmarking dataset and considering median ML parameters and average performance measures. Finally, the performances of 35 prediction models were compared, and the best model that produced the highest area under the curve (AUC), whose corresponding feature set was considered optimal, was selected.

### Selection of the Optimal Model

**Figure 4B** shows the performances of the RF-based models in terms of AUC using different feature sets, where a fluctuation was found in the initial phase, peaking in an F354-based model with an AUC of 0.801. Afterward, the AUC showed a stable performance followed by downward trend with the decrease in the number of features. Here, we selected the F354-based model as the final one owing to its best performance and named it AIPpred; its optimal ML parameters were *ntree* = 430, *mtry* = 1, and *nsplit* = 2. Interestingly, our feature selection protocol excluded most of the Trp, Cys and Met containing dipeptides and selected the remaining 354 dipeptides as optimal candidates that covered all 20 amino acids (Supplementary Table S1), which produced the best performance.

Due to the imbalanced dataset, the optimal probability cut-off value of 0.36 was chosen via grid search for AIPpred to define the class. To demonstrate the effect of our feature selection protocol, we compared AIPpred with the control (using all DPC features). **Figure 4C** shows that AIPpred MCC, accuracy, and AUC were respectively 5, 2.5, and ∼4% higher than those of the control. These results demonstrated that the numerous redundant or uninformative features present in the original feature set were eliminated through our feature selection protocol, thereby significantly improving the performance.

### Comparison of AIPpred With Other ML Algorithms

**FIGURE 5:**
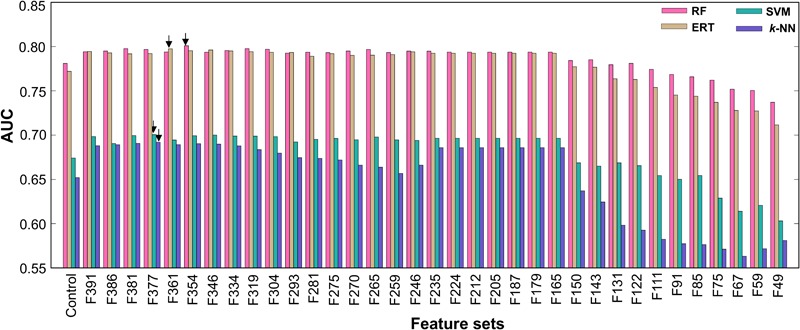
Performance of 4 different ML-based classifiers. Performance of various classifiers in distinguishing between AIPs and non-AIPs. A total of 36 classifiers (including the control) were evaluated using 10 independent 5-fold cross-validation techniques, and their average performances in terms of AUC is shown. The final selected model for each ML-based method is shown with arrows.

**FIGURE 6:**
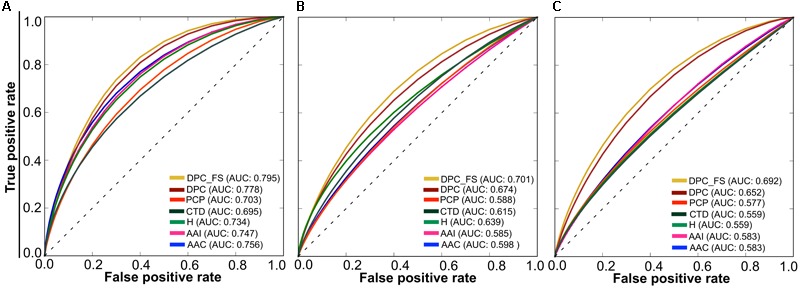
A graphical illustration to show the performance of various ML models in terms of ROC curves obtained from the 5-fold cross-validation. **(A)** ERT; **(B)** SVM; and **(C)** *k*-NN.

Generally, ML-based methods are problem specific (Dreiseitl et al., 2001; Silva et al., 2011; Khondoker et al., 2016). Hence, it is necessary to explore different ML methods on the same dataset to select the best one instead of selecting a ML method arbitrarily. In addition to RF, we also developed ERT-, *k*-NN-, and SVM-based models using the same feature selection protocol and benchmarking dataset. Each ML method has its own advantages and disadvantages (Khan et al., 2010). A detailed description of these 3 methods has been provided in our recent studies (Manavalan and Lee, 2017; Manavalan et al., 2017b). Here, the procedure of ML parameter optimization for these 3 methods, final model selection, and optimal probability cut-off value was the same as that for AIPpred. The overall performance comparison of the RF method with the other 3 methods is shown in **Figure 5**, where RF and ERT produced a similar performance regardless of the feature set used, thus indicating that ensemble-based algorithm is better suited for AIP prediction. Interestingly, the final selected model for the 3 methods (SVM, ERT, and *k*-NN) is better than that of their corresponding control (using all dipeptide composition), again emphasizing the efficiency of the feature selection protocol. We also checked whether the final selected optimal model for these 3 methods is better than other composition-based and hybrid models. **Figure 6** shows that the optimal model significantly better than their counterparts.

Finally, we compared AIPpred performance with that of the other 3 methods; the results are shown in **Table 1**, where the methods are ranked according to the AUC associated with predictive capability. The accuracy, AUC, and MCC of AIPpred were higher than those of other methods by 0.5–9%, 0.6–11%, and 1–17%, respectively. Using a *P*-value threshold of 0.05, AIPpred significantly outperformed SVM and *k*-NN, and was better than ERT, thus indicating the superiority of AIPpred. To check the transferability of AIPpred, we evaluated an independent dataset and compared it with the state-of-the-art method and 3 other ML methods developed in this study.

**Table 1:** A Comparison of AIPpred performance with other ML-based methods developed in this study using the same benchmarking dataset.

Method	MCC	Accuracy	Sensitivity	Specificity	AUC	*P*-value
AIPpred	0.460	0.734	0.758	0.711	0.801	
ERT	0.451	0.730	0.734	0.726	0.795	0.615
SVM	0.311	0.656	0.642	0.675	0.701	**<0.000001**
*k*-NN	0.291	0.641	0.512	0.770	0.692	**<0.000001**

*The first column represents the method developed in this study. The second, the third, the fourth, and the fifth respectively represent the MCC, accuracy, sensitivity, and specificity. The sixth column and the seventh represent the area under curve (AUC) and pairwise comparison of AUC between AIPpred and the other ML-based methods computed using a two-tailed *t*-test (Hanley and McNeil, 1982). A *P* ≤ 0.05 indicates a statistically meaningful difference between AIPpred and the selected method (shown in bold).*

### Performance of Various Methods on an Independent Dataset

We evaluated the performances of our 4 methods along with that of the state-of-the-art method (AntiInflam) on an independent dataset. **Table 2** shows that AIPpred achieving values of 0.479, 0.744 for MCC and accuracy, respectively. Indeed, the corresponding metrics were ∼2–28% and ∼1–17%, higher than those achieved by other methods, indicating superiority of AIPpred.

**Table 2:** Performance of various methods on independent dataset.

Method	MCC	Accuracy	Sensitivity	Specificity	AUC	*P*-value
AIPpred	0.479	0.744	0.741	0.746	0.814	
ERT	0.463	0.736	0.731	0.740	0.804	0.621
AntiInflam (MA)	0.210	0.601	0.786	0.417	0.706	**<0.000001**
SVM	0.298	0.651	0.621	0.680	0.704	**<0.000001**
*k*-NN	0.296	0.640	0.479	0.801	0.699	**<0.000001**
AntiInflam (LA)	0.197	0.575	0.258	0.892	0.647	**<0.000001**

*The first column represents the method employed in this study. The second, the third, the fourth, and the fifth respectively represent the MCC, accuracy, sensitivity, and specificity. The sixth column and the seventh represent the AUC and pairwise comparison of AUC between AIPpred and the other methods computed using a two-tailed *t*-test. A *P* ≤ 0.05 indicates a statistically meaningful difference between AIPpred and the selected method (shown in bold). In the first column, LA and MA respectively correspond to less accurate and more accurate prediction method. We note that AntiInflam LA and MA classification accuracy was computed using default threshold value of 0.5 and -0.3 (reported in Gupta et al., 2017b), respectively.*

**FIGURE 7:**
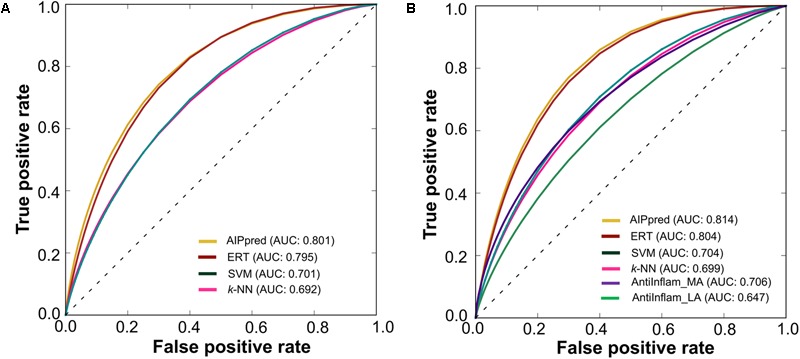
Receiver operating characteristic curves of the various prediction models. **(A)** 5-fold cross-validation on a benchmarking dataset and **(B)** independent dataset. Higher AUC values indicated better method performance.

Using a *P*-value threshold of 0.05, AIPpred significantly outperformed SVM, *k*-NN and AntiInflam suggesting its usefulness as an improvement to existing tools for predicting AIPs. Interestingly, AIPpred performed consistently well, both in training and on an independent dataset (**Figure 7**), suggesting its ability to do well in unseen peptides when compared to other ML-based models developed in this study.

### Comparison of AIPpred With the AntiInflam Method in Terms of Methodology

A detailed comparison of the differences between AIPpred and AntiInflam (Gupta et al., 2017b) in terms of methodology resulted in the following findings: (i) larger size of the benchmarking dataset used to develop AIPpred than AntiInflam. (ii) AntiInflam uses an SVM-based algorithm, whereas we explored 4 different ML-based algorithms, including SVM, and reported that the RF-based method produced the best performance, thus making AIPred the first application of an RF-based method in AIP prediction. (iii) AntiInflam uses hybrid features, whereas AIPpred uses optimal DPC features identified by the feature selection protocol. (iv) AIPred used a unique parameter-optimization procedure involving 10 independent 5-fold cross-validations to finalize the ML parameters, whereas only one 10-fold cross-validation was employed for AntiInflam.

### The AIPpred Online Prediction Server

Prediction methodologies available on a web server are practically beneficial to experimentalists, as well as to developers (Chen et al., 2013, 2017; Chen W. et al., 2016; Liu et al., 2017). A few examples of bioinformatics web servers that have been utilized for protein function predictions are available in the literature (Govindaraj et al., 2010, 2011; Manavalan et al., 2010a,b, 2011; Basith et al., 2013, 2011a). We developed an online prediction server called AIPpred.^1^ For checking the reproducibility of our findings, the datasets used in this study can be downloaded from the AIPpred web server.

## Discussion

Identifying the peptides that induce anti-inflammatory cytokines is one of the challenging task in the field of vaccine design. The computational identification of AIP candidates is essential for shortening the laborious experimental tasks. AIPs prediction is more challenging than other peptide-based prediction methods, including anticancer, antiviral and cell-penetrating peptides (Thakur et al., 2012; Tang et al., 2016; Manavalan et al., 2017a). All these methods were developed on smaller dataset with negative examples taken from randomly chosen UniProt peptides, which are not experimentally validated. However, we have used experimentally verified AIPs and non-AIPs from IEDB, whose size was ∼2-fold bigger than the dataset used in the state-of-the-art method (Gupta et al., 2017b). In general, methods developed using such experimentally verified larger dataset have a wide range of applications in modern biology (Porto et al., 2017a).

We have made a systematic attempt to understand the nature of anti-inflammatory inducing peptides and to develop the prediction model. The construction of experimentally validated nr dataset is the backbone of this study. We analyzed these peptides to understand the compositional and positional preferences of residues in AIPs, as shown in result section, Leu, Lys and Arg is highly abundant in AIPs, compared to non-AIPs. Previous studies showed that Leu-Lys rich peptides play an important role in inducing anti-inflammatory cytokines in periodontal disease (Shang et al., 2014). Furthermore, determining the biological significance of various dipeptides in anti-inflammatory induction, observed in our study, requires further studies and experimental validation.

We explored four different ML algorithms (RF, SVM, ERT, and *k*-NN) and compositional features, including AAC, AAI, DPC, CTD, and PCP for discriminating AIPs and non-AIPs. It is worth mentioning that all these ML algorithms and five different compositions were used in various sequence-based classification methods (Lata et al., 2007; Dhanda et al., 2013; Gautam et al., 2015; Gupta et al., 2017a; Manavalan et al., 2017a; Nagpal et al., 2017). Since DPC-based model from the respective algorithm produced the best performance among the different compositions, we applied a feature selection protocol on dipeptide composition and selected more important features that further improved the performance. RF produced the best performance among the various ML algorithms and named it AIPpred. Interestingly, our systematic feature selection protocol excluded most of Trp, Cys and Met containing dipeptides and selected the remaining 354 dipeptides as optimal candidates, thus indicating the arrangement of particular local ordering dipeptides plays an important role in AIPs/non-AIPs classification. Furthermore, we demonstrated that AIPpred outperformed a state-of-the-art method (AntiInflam) and 3 other methods (ERT, *k*-NN, and SVM) developed in this study when it was objectively evaluated on an independent dataset. Interestingly, AIPpred performed consistently better in benchmarking and independent datasets, suggesting its ability to predict AIPs of unseen peptides.

The improved performance of AIPpred is mainly due to the following reasons: (i) larger benchmarking dataset utilized for the model development (ii) systematic evaluation of individual composition and the selection of an appropriate composition (i.e., DPC); and applying the feature selection protocol on DPC to select the optimal feature set, which further improves prediction performance; (iii) ML parameters were obtained by a rigorous 5-fold cross-validation procedure. Here, the 5-fold cross-validation procedure was repeated 10 times, with the random portioning of the benchmarking dataset, whose median ML parameters were considered as the final one; and (iv) the choice of ML method. Interestingly, the current approach is a general one, which is applicable to numerous other peptide-based classification problems. Although AIPpred displayed a superior performance over other methods, a pressing need exists for further improvements, incorporating novel features, and exploring different feature selection techniques, including ANOVA (Zhao et al., 2017), *F*-score (Lin et al., 2017), and binomial distribution (Lai et al., 2017).

## Conclusion

The proposed predictor is quite promising in AIP prediction and available as web server at www.thegleelab.org/AIPpred. Even though AIPred represents the second publicly available method for predicting AIPs, the delivery of higher accuracy is noteworthy. Compared to experimental approaches, bioinformatics tools, such as AIPpred represent a powerful and cost-effective approach for proteome-wide prediction of AIPs. Therefore, AIPpred might be useful for large-scale AIP prediction and facilitating hypothesis-driven experimental design.

## Author Contributions

BM and GL conceived and designed the experiments and wrote the paper. BM performed the experiments. BM and TS analyzed the data. GL and MK contributed reagents/materials/software tools. All authors reviewed the manuscript and agreed to this information prior to submission.

## Conflict of Interest Statement

The authors declare that the research was conducted in the absence of any commercial or financial relationships that could be construed as a potential conflict of interest.

## Footnotes

1 www.thegleelab.org/AIPpred
