# Supplementary material for: AIPpred: Sequence-Based Prediction of Anti-inflammatory Peptides Using Random Forest
Source: Front Pharmacol. 2018 Mar 27;9:276. doi: 10.3389/fphar.2018.00276 (PMC5881105; doi:10.3389/fphar.2018.00276)
Supplement: Supplementary file 1 [file Table_1.PDF]

Supplementary information

Table S1. Dipeptides along with the FISs are shown. Yellow highlighted dipeptides were discarded and used the remaining 354 optimal features as input to RF algorithm.

S No.	Dipeptide	FISs
1	LL	0.00860
2	SL	0.00809
3	LE	0.00578
4	LI	0.00555
5	LK	0.00537
6	AL	0.00520
7	AA	0.00512
8	LS	0.00484
9	LV	0.00481
10	AV	0.00481
11	VL	0.00470
12	GS	0.00464
13	SV	0.00462
14	KL	0.00454
15	QL	0.00453
16	YL	0.00449
17	VA	0.00444
18	EA	0.00433
19	DA	0.00433
20	KV	0.00431
21	AE	0.00430
22	KA	0.00427
23	EK	0.00425
24	KK	0.00425
25	LT	0.00424
26	KG	0.00424
27	IK	0.00421
28	GA	0.00417
29	DK	0.00413
30	VT	0.00412

31	LG	0.00411
32	RL	0.00409
33	SG	0.00407
34	AG	0.00407
35	LN	0.00405
36	VD	0.00404
37	LA	0.00403
38	SE	0.00399
39	TS	0.00397
40	GL	0.00397
41	DL	0.00397
42	VS	0.00397
43	AS	0.00393
44	KS	0.00393
45	GG	0.00392
46	IL	0.00392
47	GD	0.00391
48	VV	0.00390
49	AD	0.00390
50	VK	0.00390
51	GK	0.00389
52	AK	0.00389
53	QK	0.00389
54	PS	0.00387
55	EV	0.00386
56	KP	0.00384
57	EL	0.00384
58	LD	0.00383
59	TG	0.00383
60	EF	0.00380
61	GV	0.00379
62	VE	0.00379
63	EE	0.00377
64	SA	0.00377
65	TL	0.00376

66	FL	0.00373
67	PE	0.00370
68	KE	0.00370
69	GI	0.00370
70	PA	0.00369
71	TI	0.00365
72	SK	0.00365
73	ER	0.00362
74	DV	0.00360
75	AN	0.00360
76	IP	0.00360
77	NK	0.00359
78	SS	0.00357
79	TA	0.00356
80	IA	0.00355
81	FI	0.00354
82	GE	0.00351
83	EG	0.00351
84	EN	0.00350
85	RA	0.00350
86	DE	0.00349
87	AF	0.00343
88	NL	0.00343
89	LP	0.00343
90	NA	0.00341
91	KI	0.00340
92	ID	0.00340
93	EQ	0.00339
94	TV	0.00339
95	SY	0.00338
96	TP	0.00338
97	ST	0.00338
98	ED	0.00337
99	KD	0.00336
100	VI	0.00335

101	AT	0.00335
102	YK	0.00334
103	FK	0.00334
104	FA	0.00334
105	AI	0.00334
106	SP	0.00333
107	NV	0.00333
108	VG	0.00332
109	DG	0.00332
110	EI	0.00331
111	IS	0.00330
112	GT	0.00330
113	TD	0.00330
114	LR	0.00330
115	IT	0.00329
116	YD	0.00327
117	SI	0.00326
118	PL	0.00326
119	KY	0.00325
120	AP	0.00323
121	TE	0.00322
122	DD	0.00321
123	RS	0.00319
124	KM	0.00319
125	AR	0.00318
126	KT	0.00317
127	TT	0.00317
128	LF	0.00317
129	LQ	0.00316
130	IV	0.00314
131	KF	0.00310
132	FE	0.00310
133	QQ	0.00310
134	IE	0.00309
135	KR	0.00309

136	VR	0.00309
137	GR	0.00307
138	PV	0.00306
139	RI	0.00305
140	RV	0.00305
141	ES	0.00304
142	DQ	0.00303
143	NG	0.00303
144	GF	0.00296
145	NN	0.00295
146	FT	0.00294
147	VP	0.00294
148	TK	0.00292
149	IQ	0.00291
150	VY	0.00291
151	MA	0.00290
152	RR	0.00289
153	QA	0.00288
154	II	0.00287
155	RG	0.00286
156	DP	0.00286
157	IG	0.00284
158	ET	0.00284
159	SN	0.00284
160	DS	0.00283
161	NI	0.00282
162	DI	0.00282
163	GN	0.00282
164	IR	0.00281
165	SD	0.00281
166	PG	0.00278
167	VN	0.00278
168	GP	0.00278
169	KN	0.00278
170	PT	0.00277

171	SR	0.00276
172	FD	0.00276
173	IN	0.00275
174	YS	0.00275
175	VF	0.00274
176	PN	0.00274
177	QE	0.00273
178	FN	0.00272
179	NP	0.00271
180	SF	0.00269
181	PK	0.00268
182	YT	0.00266
183	TY	0.00264
184	FV	0.00264
185	LY	0.00263
186	VQ	0.00263
187	YE	0.00262
188	DT	0.00260
189	NS	0.00259
190	GY	0.00258
191	AM	0.00258
192	YY	0.00257
193	NE	0.00257
194	EP	0.00256
195	RK	0.00255
196	YV	0.00255
197	HV	0.00254
198	QV	0.00254
199	NF	0.00252
200	TF	0.00252
201	DN	0.00252
202	DY	0.00252
203	QI	0.00251
204	AY	0.00251
205	YA	0.00251

206	RT	0.00249
207	NY	0.00248
208	SQ	0.00247
209	IF	0.00243
210	IY	0.00243
211	YI	0.00243
212	DR	0.00240
213	RP	0.00240
214	PI	0.00240
215	RE	0.00238
216	AQ	0.00238
217	PF	0.00238
218	FS	0.00236
219	NT	0.00234
220	QG	0.00233
221	TQ	0.00232
222	WV	0.00231
223	FG	0.00231
224	QS	0.00230
225	KQ	0.00230
226	QR	0.00229
227	EM	0.00229
228	EY	0.00228
229	YF	0.00226
230	YG	0.00226
231	YP	0.00225
232	LM	0.00225
233	PD	0.00224
234	PR	0.00222
235	RD	0.00221
236	YQ	0.00219
237	PP	0.00219
238	HA	0.00218
239	PQ	0.00218
240	FR	0.00217

241	FF	0.00214
242	PY	0.00214
243	TN	0.00212
244	FY	0.00212
245	QT	0.00211
246	RF	0.00211
247	EH	0.00210
248	RN	0.00210
249	NQ	0.00209
250	DF	0.00208
251	ND	0.00208
252	QP	0.00208
253	HL	0.00206
254	GH	0.00206
255	YN	0.00204
256	NR	0.00204
257	GQ	0.00204
258	AH	0.00204
259	QD	0.00202
260	TR	0.00199
261	HP	0.00199
262	KC	0.00196
263	MV	0.00195
264	HE	0.00195
265	MQ	0.00193
266	SH	0.00190
267	FQ	0.00186
268	QF	0.00182
269	CS	0.00182
270	YR	0.00181
271	VH	0.00179
272	FP	0.00176
273	ML	0.00176
274	HG	0.00176
275	ME	0.00170

276	MK	0.00168
277	GM	0.00166
278	QN	0.00164
279	QY	0.00162
280	CG	0.00161
281	MT	0.00161
282	MG	0.00160
283	HF	0.00159
284	VM	0.00158
285	RQ	0.00156
286	IM	0.00155
287	HI	0.00154
288	MD	0.00153
289	KH	0.00153
290	FH	0.00153
291	MI	0.00153
292	IH	0.00150
293	HS	0.00150
294	HD	0.00150
295	MF	0.00150
296	AW	0.00149
297	EC	0.00146
298	WD	0.00146
299	SM	0.00143
300	HT	0.00142
301	PC	0.00141
302	CF	0.00141
303	TC	0.00141
304	RY	0.00141
305	LH	0.00140
306	CE	0.00139
307	NH	0.00136
308	RH	0.00136
309	WL	0.00136
310	MS	0.00135

311	DH	0.00135
312	HW	0.00134
313	HR	0.00134
314	DM	0.00133
315	VC	0.00133
316	CT	0.00133
317	AC	0.00132
318	GC	0.00132
319	CK	0.00131
320	CV	0.00129
321	VW	0.00129
322	RM	0.00127
323	NM	0.00127
324	CL	0.00127
325	QH	0.00125
326	HN	0.00125
327	TH	0.00124
328	CP	0.00124
329	YM	0.00124
330	PH	0.00124
331	IC	0.00123
332	MP	0.00123
333	HK	0.00122
334	SC	0.00122
335	RC	0.00119
336	SW	0.00117
337	WI	0.00116
338	TW	0.00116
339	MR	0.00116
340	CH	0.00115
341	WE	0.00115
342	WA	0.00114
343	WR	0.00113
344	LC	0.00112
345	MN	0.00112

346	PM	0.00110
347	HY	0.00109
348	HM	0.00108
349	CA	0.00108
350	DC	0.00105
351	WM	0.00104
352	TM	0.00103
353	CC	0.00100
354	CM	0.00100
355	WY	0.00098
356	CD	0.00097
357	CN	0.00096
358	YH	0.00094
359	CI	0.00094
360	FC	0.00092
361	NW	0.00090
362	NC	0.00090
363	DW	0.00089
364	QW	0.00089
365	YC	0.00088
366	WC	0.00087
367	EW	0.00087
368	HQ	0.00086
369	GW	0.00086
370	WQ	0.00085
371	IW	0.00084
372	QM	0.00084
373	KW	0.00083
374	WG	0.00083
375	MY	0.00082
376	WT	0.00082
377	FM	0.00080
378	HH	0.00079
379	MH	0.00078
380	FW	0.00070

381	LW	0.00070
382	PW	0.00068
383	WS	0.00066
384	CQ	0.00066
385	WP	0.00063
386	MM	0.00062
387	CR	0.00060
388	CY	0.00057
389	WN	0.00056
390	RW	0.00056
391	HC	0.00050
392	MC	0.00049
393	WK	0.00049
394	QC	0.00047
395	YW	0.00045
396	WF	0.00038
397	MW	0.00021
398	WH	0.00018
399	CW	0.00017
400	WW	0.00011
